# Distinctive CD26 Expression on CD4 T-Cell Subsets

**DOI:** 10.3390/biom11101446

**Published:** 2021-10-02

**Authors:** Oscar J. Cordero, Carlos Rafael-Vidal, Rubén Varela-Calviño, Cristina Calviño-Sampedro, Beatriz Malvar-Fernández, Samuel García, Juan E. Viñuela, José M. Pego-Reigosa

**Affiliations:** 1 Department of Biochemistry and Molecular Biology, Campus Vida, University of Santiago de Compostela, 15782 Santiago de Compostela, Spain; ruben.varela@usc.es (R.V.-C.); criscalsa@gmail.com (C.C.-S.); 2 Rheumatology & Immune-Mediated Diseases Research Group (IRIDIS), Galicia Sur Health Research Institute (IISGS), SERGAS-UVIGO, 36312 Vigo, Spain; carlos.rafael@iisgaliciasur.es (C.R.-V.); beatriz.malvar@iisgaliciasur.es (B.M.-F.); samuel.garcia@iisgaliciasur.es (S.G.); jose.maria.pego.reigosa@sergas.es (J.M.P.-R.); 3 Rheumatology Department, University Hospital Complex of Vigo-SERGAS, 36312 Vigo, Spain; 4 Service of Immunology, University Hospital Complex of Santiago de Compostela-SERGAS, 15782 Santiago de Compostela, Spain; juan.vinuela.roldan@sergas.es

**Keywords:** soluble CD26, T cell memory, T helper polarization, DPP4

## Abstract

Immune system CD4 T-cells with high cell-surface CD26 expression show anti-tumoral properties. When engineered with a chimeric antigen receptor (CAR), they incite strong responses against solid cancers. This subset was originally associated to human CD4 T helper cells bearing the CD45R0 effector/memory phenotype and later to Th17 cells. CD26 is also found in soluble form (sCD26) in several biological fluids, and its serum levels correlate with specific T cell subsets. However, the relationship between glycoprotein sCD26 and its dipeptidyl peptidase 4 (DPP4) enzymatic activity, and cell-surface CD26 expression is not well understood. We have studied ex vivo cell-surface CD26 and in vitro surface and intracellular CD26 expression and secretome’s sCD26 in cultured CD4 T cells under different polarization conditions. We show that most human CD26negative CD4 T cells in circulating lymphocytes are central memory (T_CM_) cells while CD26high expression is present in effector Th1, Th2, Th17, and T_EM_ (effector memory) cells. However, there are significant percentages of Th1, Th2, Th17, and Th22 CD26 negative cells. This information may help to refine the research on CAR-Ts. The cell surface CD45R0 and CD26 levels in the different T helper subsets after in vitro polarization resemble those found ex vivo. In the secretomes of these cultures there was a significant amount of sCD26. However, in all polarizations, including Th1, the levels of sCD26 were lower (although not significantly) compared to the Th0 condition (activation without polarization). These differences could have an impact on the various physiological functions proposed for sCD26/DPP4.

## 1. Introduction

Human CD4 cells with high CD26 expression have an enhanced chemokine receptor profile and stemness, are cytotoxic and resistant to apoptosis [1]. This may have clinical consequences in oncology, where CD26high T cells engineered with a chimeric antigen receptor (CAR) ablated large human tumors to a greater extent than subsets enriched in Th17, Th1, or Th2 cells [2]. Recent works described that CD4 CD26high T cells are composed of either Th1, Th17, or hybrid Th1/Th17 cells with the capacity for trans-endothelial migration [3,4,5,6,7,8], and its presence correlated with clinical severity in multiple sclerosis [5] and rheumatoid arthritis [7].

The first analyses on CD26 expression on CD4 lymphocytes showed a correlation with helper T cells bearing an effector/memory phenotype as defined by different CD45R isoforms [9,10], However, since (i) all CD4+ CD8+ medullary thymocytes express CD26 [11], (ii) 90% of human cord blood T cells, which are almost entirely CD45RA+, are also CD26+ [12], and (iii) the frequency of CD26+ T cells is much lower in adult blood and within lymphoid tissue [12,13], all this suggests that CD26 expression can also be suppressed as T cells differentiate. In fact, subsets of CD4 or CD8 CD45R0 CD26neg (negative) T cells with clinical implications have been identified [5,7,14,15,16], including Tregs [16].

CD26 is a multifunctional glycoprotein present on the cell surface of many epithelial cells in tissues, not only in circulating T lymphocytes, and also as a soluble form (sCD26) in biological fluids [17,18,19,20]. CD26 belongs to the subgroup of prolyl oligopeptidases (dipeptidyl peptidase 4, DPP4, EC 3.4.14.5) and through its N-terminal X-Pro enzymatic cleaving activity regulates chemotactic responses to the inflammatory chemokines CCL, 3–5, 11 and 22, and CXCL, 2 and 9–12 [1,5,6] and other biologically active peptides such as NPY, VIP, or incretins [18,20]. CD26 was implicated in the regulation of immune functions also because CD26 participates in T cell infiltration, at least in adhesion through its binding to collagen and fibronectin in the extracellular matrix (ECM), and to adenosine deaminase (ADA) and integrin beta-1 in other cell types [18,19,20,21]. In addition, certain anti-CD26 monoclonal antibodies (mAbs) were able to transmit an activating signal to the T cell [9].

Some functions have been proposed for sCD26 in addition to its proteolytic activity, as a ligand of the protease-activated G protein-coupled receptor (PAR2) with clinical consequences in inflammatory diseases including infection, autoimmune [22,23,24] and probably cancer [25,26,27,28], or as a ligand of caveolin-1 in antigen presenting cells [29,30].

We and others showed correlations between the relatively high levels of DPP4 enzymatic activity and/or sCD26 serum levels with specific T cell subsets [7,15,17]. Most data suggest that CD26 is shed from the cell surface [20], although a mechanism of secretion cannot be excluded (rev in [17,18]), and CD26 has been frequently found in exosomes [31] and present in secretory lysosomes and granules of several T lymphocyte populations including CD4 [32,33]. However, the circumstances that cause altered levels in many diseases are unknown in most cases (reviewed in [18,19]).

We have studied here the in vitro relationship between sCD26 and cell surface CD26 in different T cell populations, which were described ex vivo. This relationship must be clarified because both are therapeutic targets [17,34,35] and clinical biomarkers [18,19].

## 2. Materials and Methods

### 2.1. Biological Samples

Healthy donors were recruited from the Agency for the Donation of Organs and Blood (ADOS, Santiago de Compostela, Spain) with the approval of the Director of the Agency and the Clinical Research Ethics Committee of Galicia.

For serum collection, peripheral venous blood was collected using BD SST II *Advance* tubes (BD Biosciences, Madrid, Spain) and allowed to clot at room temperature and centrifuged at 2000× *g* for 15 min. Serum was stored at −80 °C until use.

Blood cells were collected using TransFix Vacuum Blood Collection Tubes (Cytomark, Buckingham, UK) if stored at 4 °C, or BD Vacutainers (BD Biosciences), Madrid, Spain) if used directly in flow cytometry or processed for cell culture.

### 2.2. Ethics Statement

All the procedures described were performed according to clinical ethical practices of the Spanish and European Administrations and approved by the Local Ethics Committee (Comité Ético de Investigación Clínica de Galicia, Xunta de Galicia, code 2010/298). Written informed consent was obtained from all participants.

### 2.3. Flow Cytometry Analysis

For tetracolor flow cytometry determinations of CD26 expression on T cells, routine protocols have been used [10]. Peripheral blood mononuclear cells were stained with an optimized mix of anti-CD3 (clone 33-2A3)/CD4 (clone HP2/6)/CD45R0 (clone UCHL-1)/CD26 (clone TP1/19) antibodies (or mouse IgG1 and IgG2a isotype controls, clones B11/6 and B12/8, Immunostep, Salamanca, Spain) in PBS containing 1% BSA and 0.05% sodium azide (FACS buffer) and incubated at 4 °C for 30 min.

First, different subsets of CD4+ T cells were classified according to their expression of CD26 (anti-CD26-FITC and –PE, Immunostep, Salamanca, Spain), and CD45R0 as a marker for effector/memory subsets [7,10]. Th17 and Th22 subsets were characterized by staining with combinations of anti-CD4-APC, anti-CD45R0, anti-CD161-PE (clone DX12), and anti-CD194 (CCR4)-PerCP-Cy5.5 (clone 1G1, BD Biosciences), anti-CD196 (CCR6)-FITC (clone R6H1, eBioscience) and anti-CCR10-PE (clone 314305, R&D systems), as described [10].

For central (CM) and effector memory (EM) phenotyping as described previously [7,21], antibody combinations of anti-CD4-APC, anti-CD45R0 and anti-CD26 with CCR7 (clone 2-L1-A), CD62L (clone SK11), CD27 (clone 0323), CXCR5 (clone 2G8), CCR4, CXCR3 (clone 1C3/CXCR3) or CCR5 (clone 2D7/CCR5) stainings (all from BD Biosciences, Madrid, Spain) were studied.

For intracellular staining, cells were fixed and permeabilized with the the BD Biosciences Cytofix/Cytoperm Kit following the manufacturer’s protocol.

Cells were acquired using a Becton-Dickinson FACScalibur and analyzed with the Flowing Software program (Perttu Terho, Turku Centre for Biotechnology, Finland, EU) or FCSalyzer (Sven Mostböck, http://sourceforge.net/projects/FCSalyzer, accessed on 1 October 2021).

### 2.4. Cell Culture and Polarization

PBMCs were isolated from whole blood of healthy donors using Ficoll density gradient centrifugation (GE Healthcare, Barcelona, Spain). Naïve CD4 T cells were purified using the Naïve CD4 T Cell Isolation Kit II (Miltenyi Biotec, Madrid, Spain) according to the manufacturer’s protocol. The percentage of naïve CD4 T cells obtained from different individuals ranged from 4.7% to 14.7% (median 9.8%). The purity of this naïve cell population was assessed by flow cytometry (94.2% or higher were CD3+ CD4+, and 85.4% or higher were CD45RA^+^ with less than 3.3% of CD45R0^+^ cells; data not shown).

Naïve CD4 cells were seeded in serum free media (AIM-V, Invitrogen) to avoid the influence of exogenous molecules present in fetal calf serum (FCS) and stimulated with anti-CD3/CD28 beads (Dynabeads T cell expander, Invitrogen) at one bead per cell. To polarize the response, antibodies and cytokines were added to the culture media at the beginning of the stimulation period or left untouched (Th0 response). Those conditions were for Th1, IL-12 (2 ng/mL) and anti-IL-4 neutralizing antibody (100 ng/mL); for Th2, IL-4 (25 ng/mL) and anti-IL-12 neutralizing antibody (2 µg/mL); and for Th17, IL-1β (10 ng/mL), IL-23 (10 ng/mL), anti-IL-4 neutralizing antibody (1 µg/mL) and anti-IFN-γ neutralizing antibody (1 µg/mL). All cytokines were purchased from Peprotech (Peprotech, London, UK), with the exception of IL-23, which was purchased from EBioscience (EBioscience, Madrid, Spain); neutralizing antibodies were obtained from BD Pharmingen (BD Biosciences, Madrid, Spain). Proof that naïve CD4 T cells are correctly directed into the Th1, Th2 or Th17 phenotypes had been previously determined by the levels of cytokines characteristic for each T helper subset (IFN-γ, IL-13 and IL-17A, respectively), and the RNA expression analysis for the corresponding transcription factors (T-bet, GATA3 and ROR-C2, respectively) [36].

After 72 h of stimulation, cells were collected by centrifugation and culture supernatants stored at −20 °C for use in subsequent sCD26 determination.

### 2.5. Measurement of Soluble CD26 Protein

As described previously [10,35], the sCD26 concentration was measured with the human DPPIV/CD26 DuoSet ELISA development System kit (RnD Systems) according to the manufacturer’s instructions (the limit of detection specified is 20 pg/mL). All samples were measured in duplicate in 96-well Corning plates. The wells were first covered with 50 µL of the capturing antibody (2 ng/µL) in PBS and allowed to incubate overnight at room temperature. These wells were blocked for two hours with 300 µL/well of PBS, 3% BSA before 25 µL of each secretome’s samples were mixed with 25 µL of PBS 0.5% BSA (50 µL/well), and then the plate was incubated for 90 min. The same with the revealing antibody (50 μL/well) before adding the same volume of streptavidin [1:100] in PBS, 0.5% BSA, incubated for 30 min. Finally, OPD substrate (o-phenylenediamine dihydrochloride, Sigma OPD Fast, MerckSigmaAldrich) (100 μL/well) was added and incubated for 30 min before the absorbance reading at a wavelength of 450 nm. Between each step, 6 repetitions of washing were carried out with 200 μL/well of PBS, 0.05% Tween.

### 2.6. Statistical Analysis

Descriptive statistics were obtained for continuous (mean and SD) and categorical variables (frequencies). Differences in sCD26 protein concentration, percentages, and mean or median intensity of fluorescence of cell surface markers between groups were assessed using the parametric Student’s *t* test or the nonparametric Mann-Whitney *U* test. The one-way ANOVA test was carried out to compare the variables among more than two groups. The post-hoc HSD Tukey analysis was done with equality of variances and the T3 Dunnet test without equality of variances. Pearson correlation was used to evaluate the strength of the linear relationship between the measured variables. *p*-values < 0.05 were considered statistically significant. Statistical analyses were carried out with the software SPSS version 20 (SPSS, Chicago IL, USA).

## 3. Results

### 3.1. Relationship between the Cell Surface CD26 and CD45R0 Isoform in Human Peripheral Blood CD4 T Lymphocytes

**Figure 1:**
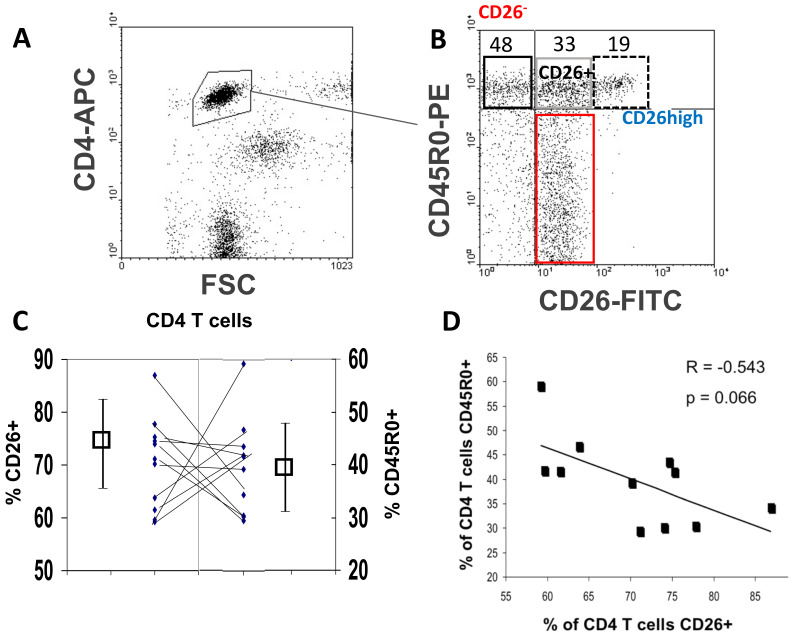
Cell-surface CD45R0 and CD26 in the CD4 T cells. (**A**) Representative (*n* = 11) flow cytometry dot-plot showing lymphocytes gated physically on FSC and CD4 (controls are shown in Supplemental Figure S1). (**B**) Dot-plots showing the differential expression of CD45R0 and CD26 in the lymphocyte region gated in A: CD4+ CD45R0low/ − CD26+ (naïve T cells; red square); and effector/memory CD4+ CD45R0+ CD26− (CD26neg; black square, mean ± SD 47.5 ± 12.0% of CD45R0+ ; range 33–72.2%), CD4+ CD45R0+ CD26+ (intermediate; grey square) and CD4+ CD45R0+ CD26++ (CD26high; dotted black square, 18.9 ±6.7% of CD45R0+ ; range 5–28.5%). (**C**) Matching of CD45R0+ cells (mean of % values ± SD, range 29.5–59.2%) and CD26+ (range 59.2–86.9%) CD4 lymphocytes in each healthy donor (*n* = 11). (**D**) Analysis of correlation between percentages of CD45R0+ and CD26+ in CD4 lymphocytes (Pearson’s correlation).

CD4 T memory cells and many effector cells bear the isoform CD45R0 phenotype [4,5,6,7,10,11,12,13] and supposedly CD45R0 and CD26 are up-regulated in the memory/effector CD4 T cell subpopulation [8,10,11,12,18]. Ex vivo, in peripheral blood obtained from 11 healthy donors, with CD45R0 positivity ascribed to those cells with high anti-CD45R0 mAb staining in the whole CD4 population (the cells with low CD45R0 staining were ascribed to naïve T cells), (Figure 1, panels A and B), the mean ± SD of CD45R0+ percentages was 39.9 ± 8.8% and of CD26+ was 70.4 ± 8.6. 

Outliers above and below of cutoff values defined from mean + 1 SD and mean − 1 SD, respectively, were the same number for CD45R0 and CD26, 1/11 above and 3/11 below. However, they did not match and in the only one sample with both outliers, the value of CD45R0 was above and of CD26 was below the cutoffs (Figure 1C). In fact, the positivity values of both markers in the CD4 population showed a negative correlation trend (Figure 1D).

The CD26high population was defined from the limit of CD26 staining in the remaining CD4 CD45R0− population and the Figure 1B shows the four different T cell subsets gated as in [4], CD45R0 CD26neg, CD45R0 CD26+ (standard), CD45R0 CD26high, and CD45R0− CD26+ (mostly naïve) cells. The expression of CD26 in the latter population (which includes the CD45R0low cells) was 81.7 ± 5.0%, much higher than that of the CD4 CD45R0 population, 52.5 ± 12%. This is explained because the CD4 CD45R0 population is enriched with CD26neg cells (Figure 1B, black square), reaching almost 50% of the memory/effector cells. This subset is larger than the better-known CD45R0 CD26high population (19%, Figure 1B, doted square), also present in CD8 cells (data not shown), which has been rarely studied quantitatively in a physiological context [3,4,5,8,9], leaving around 30% of CD45R0 lymphocytes with the intermediate expression of CD26 (Figure 1B, grey square), like that of the naïve CD4 cells (Figure 1B, red square). According to the mean of fluorescence intensity (MFI), the CD26high subset is expressing 3 to 6 times more CD26 than this intermediate CD26+ population, in coherence with previously published data [3].

Obviously, these results reject that both proteins are up regulated in all the memory/effector CD4 T cells.

### 3.2. Relationship between the Cell-Surface CD26 and the Phenotype of Central (CM), Effector/Memory (EM), and Treg CD4 T Cell Subsets

The T_CM_ and T_EM_ (including effector) subsets can be identified by the surface expression of CD45R0, CCR7, CD62L, and CD27 in T_CM_ and the downregulation of these molecules in T_EM_ [6,37].

**Figure 2:**
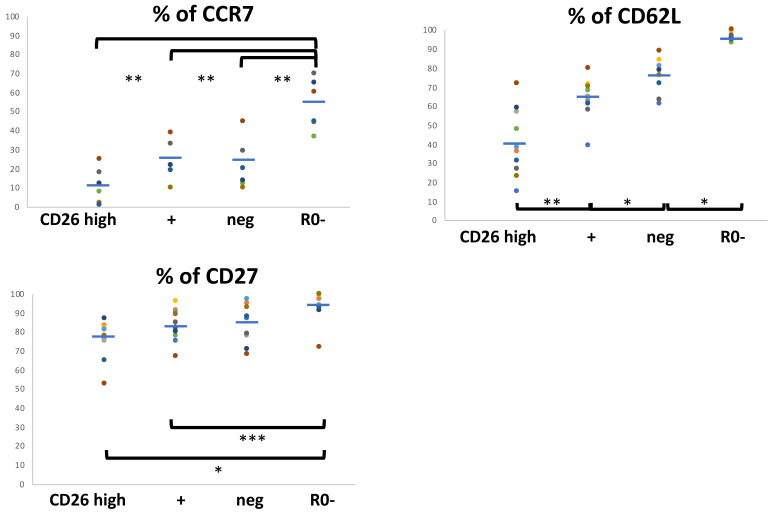
Phenotypes of central (CM) and effector/memory (EM) in CD4 T cell subsets defined with CD26 and CD45R0. Surface CD27, CD62L and CCR7 positivity frequencies in the four CD4^+^ T cell subsets defined by cell surface CD45R0 and CD26. Lymphocytes were gated using the same strategy shown in Figure 1, and for each marker of T_EM_ and T_CM_ subsets, its panel shows the respective frequencies in the respective gated region (the three CD45R0 with CD26high, + or neg, and the R0-, mean of % values ± SD respectively: CD27+, 76.8 ± 10, 83.1 ± 8.3, 85.3 ± 10.7, 94.5 ± 8.2, *n* = 11; CD62L +, 40.4 ± 17.1, 63.6 ± 10.2, 76.5 ± 8.4, 96.5 ± 2, *n* = 11; CCR7 + , 11 ± 9.4, 23.7 ± 10.5, 21.7 ± 13.3, 53.5 ± 13.3, *n* = 6). * *p* < 0.001, ** *p* < 0.01, *** *p* < 0.05.

The expression of CCR7 was very variable among individuals (range 1–45% and 37–70% of the CD45R0 and CD45R0− cells, respectively). There was no clear correlation between anti-CD26 and anti-CCR7 staining but most CD26high cells are CCR7− and more, but not many, CD26neg and CD26+ cells are CCR7+ (Figure 2). 

In fact, there are more CCR7− CD26neg than CCR7− CD26high cells (considering the results of the previous section, and Supplementary Figure S1B, left part of the histogram).

CD62L (L-selectin) positivity showed a slight less variability (range 36–85% of the CD45R0 cells) and its correlation with CD26 was higher. More than 60% and 70% of the CD26+ and CD26neg subsets cells retain CD62L, in contrast to a lower 40% in the CD26high (in disagreement with previous reports [6]) (Figure 2). Still, the total frequency of CD62L− CD26neg cells is higher than CD62L− CD26+ or CD26high subsets (considering the results of the previous section, and Supplementary Figure S1C, left part of the histogram).

Finally, regarding CD27, most CD45R0 cells maintains its expression, and CD27− cells are found in the CD26high, CD26+ and CD26neg subsets in similar (23 to 15%) frequencies, but the majority of CD27− cells are CD26neg (considering the results of the previous section, and Supplementary Figure S1D).

In the case of regulatory T cells (Tregs) cell surface CD26 was compared only with the expression of CD25 [16]. Around a 60% (range 58–65%) of the CD45R0 cells are CD25 +, including a 10% of CD45R0 lymphocytes being CD25high (Supplementary Figure S2). Most CD26+ and CD26high cells are CD25+, and no CD26high cell is CD25high. As expected, [1,16], an important percentage of CD25high cells are CD26neg (Supplementary Figure S2, histogram, left circle), but there is a subset of CD25high CD26+ cells (Supplementary Figure S2, histogram, right circle). Finally, a significant but variable percentage of the CD25− lymphocytes are also CD26neg.

Summarizing, the CD26high cells are mostly T_EM_ because of CCR7, CD62L and CD27 downregulation in a large percentage (but not all) of these cells. Likewise, more CD26neg and CD26+ (intermediate) lymphocytes are related with the CCR7+ CD27+ CD62L+ T_CM_ population, the main difference with the naïve T cells, which also express these markers, being the higher CCR7+ in the latter. However, there is a CD26neg population showing either a variable or negative expression of CCR7, CD62L, and CD27, as with the CD26high cells. Also, almost but not all Tregs are CD26neg.

### 3.3. Functional Programs in CM and EM Human T CD4 Cell Subsets in Relation to the Cell-Surface CD26

T_CM_ and T_EM_ subsets with distinct functional programs can be identified according to the expression of cell surface chemokine receptors CXCR5, CCR4, CXCR3, and CCR5 [37].

**Figure 3:**
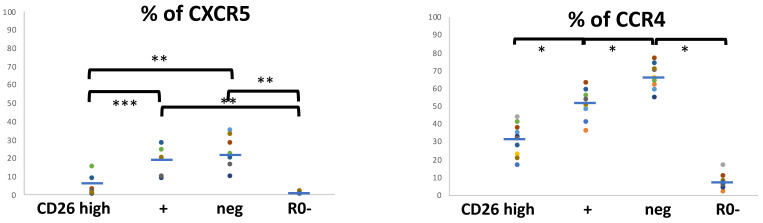
Markers of pre-effector programs in CD4 T cell subsets defined with CD26 and CD45R0. Cell-surface CXCR5 and CCR4 positivity frequencies in the four CD4^+^ T cell subsets defined by surface CD45R0 and CD26 expression. Lymphocytes were gated using the same strategy shown in Figure 1, and for each marker of T_EM_ and T_CM_ subsets, its panel shows the respective frequencies in the respective gated region (the three CD45R0 with CD26high, + or neg, and the R0-, CXCR5: 5.6 ± 5.5, 19.9 ± 7.8, 23.4 ± 9.1, 0.6 ± .8, *n* = 7; CCR4: 31.4 ± 8.4, 51.5 ± 7.7, 65.2 ± 7.3, 6.6 ± 4.3, *n* = 11). * *p* < 0.001, ** *p* < 0.01, *** *p* < 0.05.

The expression of chemokine receptor CXCR5 marks non-polarized T_CM_ lymphocytes. In coherence to the results above, almost all the CD26high cells are CXCR5− and an important percentage of CXCR5+ cells are CD26neg or CD26+ (Figure 3 and Supplementary Figure S3A). However, most CD26+ and CD26neg are also CXCR5−.

Regarding CCR4, marker of T_CM_ pre-effector cells, Th2 and other phenotypes (see below), it was found in around 60% of the CD45R0 cells (range 54–65%) and also some CD45R0- cells (7%). A significant percentage of CD26high cells are CCR4–, CD26neg cells mostly CCR4+ and CD26+ cells around half were CCR4+ (Figure 3). In addition, the presence of a CD26neg cell population that overexpresses CCR4 can be observed (Supplementary Figure S3B, circle).

**Figure 4:**
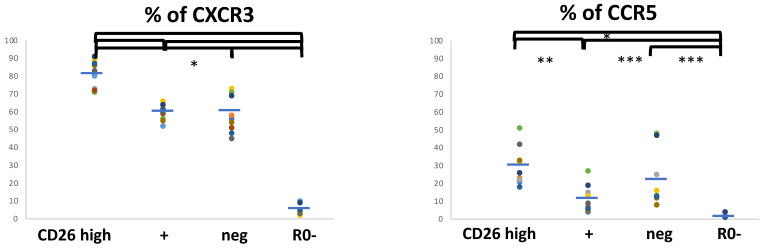
Markers of effector programs in CD4 T cell subsets defined with CD26 and CD45R0. Cell surface CXCR3 and CCR5 positivity frequencies in the four CD4+ T cell subsets defined by surface CD45R0 and CD26 expression. Lymphocytes were gated using the same strategy shown in Figure 1, and for each marker of T_EM_ and T_CM_ subsets, its panel shows the respective frequencies in the respective gated region (the three CD45R0 with CD26high, + or neg, and the R0-, CXCR3: 81.5 ± 6.9, 58.9 ± 4.6, 60.6 ± 10.6, 5 ± 2.4, *n* = 11; CCR5: 29.8 ± 10.9, 11.7 ± 7.6, 21.1 ± 15.8, 1.7 ± 1, *n* = 10). * *p* < 0.001, ** *p* < 0.01, *** *p* < 0.05.

CXCR3 is a marker of T_CM_ pre-effector Th1 and Th2 and also T_EM_ Th1 and Th2 cells. Around 65% of CD45R0 cells (range 58–80%) was CXCR3+. These data mean that some CD45R0 cells must co-express CXCR3 and CCR4. Most, but not all, of the CD26high subset are CXCR3+ as well as a minor percentage of CD45R0- cells (the naïve T cell subset) (Figure 4). 

In coherence with the results above, many CD26neg and CD26+ cells are also CXCR3+ (Figure 4), as well as half CXCR3- cells are CD26neg (Supplementary Figure S4C). Incidentally, both CD26+ and CD26high expressing CXCR3+ cells show lower mean intensity (Supplementary Figure S3C, black and blue lines respectively) compared to the CXCR3 staining intensity seen in CD26neg cells (Supplementary Figure S3C, red line).

CCR5 is a marker of T_EM_ Th1 phenotypes. As shown in Supplementary Figure S3D, many CCR5+ cells (around 50%) are CD26high and, although not observed with the same intensity in all cases, many CD26neg cells express lower levels of CCR5. But only 30% and 20% of CD26high and CD26neg are CCR5+ (Figure 4).

Summarizing, although many CD26high T_EM_ cells show a Th1 phenotype (CXCR3+ CCR5+), the existence of a minor CD26high T_EM_ population (around 20%) with a Th2 phenotype can be confirmed (CCR4+ CXCR3- CCR5- CXCR5-). Likewise, there are similar subsets in the CD26neg cells.

### 3.4. Relationship between CD26 and Th17 and Th22 CD4 Subsets

**Figure 5:**
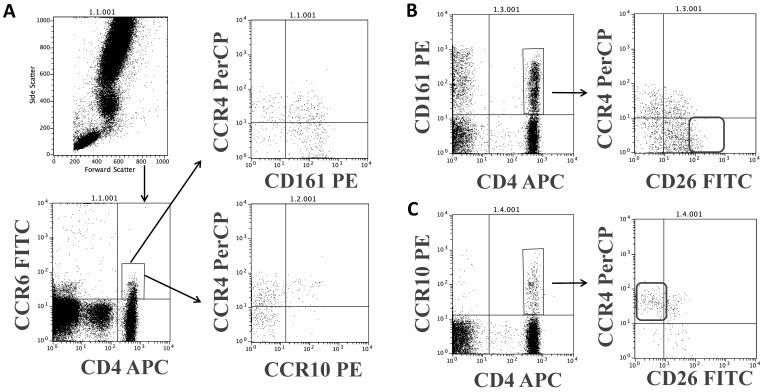
Cell-surface CD26 in T helper 17 and 22 cell subsets defined by CD4, CCR6 (CD196), CD161 and CCR4 (CD194). (**A**) Representative flow cytometry (*n* = 4) dot-plots showing lymphocyte gating strategy for CD4, CCR6, and CD161 vs. CCR4 for Th17 cells (above) and CCR10 vs. CCR4 for Th22 cells (below). (**B**) In Th17 cells, the expression of CD26 in the CCR4+ cells is low and the CD26high cells (if any) are CCR4- (the square). (**C**) Most Th22 cells are CCR4+ and CD26- (the square) or CD26low.

Th17 and Th22 proinflammatory cells, implicated in the pathogenesis of many autoimmune diseases, cancer and more, are a very minor part of the CCR4+ CD4 cells and are CD45R0 [1,37,38,39,40,41]. The expression of CD4, CCR4, CCR6, and CD161 (for Th17) or CCR10 (for Th22) can identify these subsets [7]. We found that Th17 CCR4+ cells are a mix of CD26+ and CD26neg cells (Figure 5B, left dot plot). 

The CD26high subset (a minor part) is CCR6+ and CD161+ as described [10] but CCR4- (Figure 5 B, square). In addition, Th22 lymphocytes are mostly CCR4+ and CD26neg or CD26low (Figure 5 C, square).

### 3.5. Cell-Surface CD26 in In Vitro Polarized CD4 T Lymphocytes

**Figure 6:**
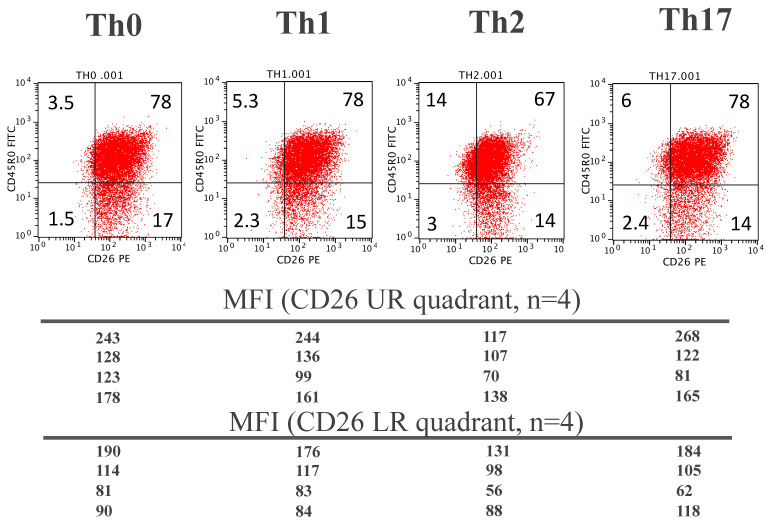
Cell surface CD45R0 and CD26 staining in CD4 T cells activated in-vitro from naïve cells under different polarization conditions (controls are shown in Supplemental Figure S6): Th0 (no polarization), Th1, Th2 and Th17. Numbers in each quadrant correspond to the respective percentages. The dot plots are representative from one out of 4 different experiments. For comparison, MFI data from CD26 staining are shown in the bottom for each condition (as the range of intensities from one experiment to another was somewhat high, the means were not calculated).

We next evaluated whether naïve CD4 T cells polarized in-vitro to either a Th1, Th2, or Th17 phenotype or non-polarized Th0 [36] would show similar CD26 expression patterns than the ex-vivo analysis of PBMC. Figure 6 shows a representative result (*n* = 4) of cell surface expression of both CD45R0 and CD26 in the different T helper subsets. 

A trend to more CD26high cells can be observed in all conditions, in particular Th1 and Th17 (Figure 6). In all the polarization conditions some cells are CD26neg, particularly in the Th2 and Th17 conditions (Figure 6, see MFI values). The downregulation of CD26 did not reach the levels seen in the ex-vivo analysis, probably due to the short culture period (3 days).

The levels of intracellular CD26 staining in the different Th subsets polarized in-vitro were also evaluated. For a good comparison, only CD45R0+ blasts were gated. In these conditions, intracellular CD26 levels (mean, and particularly median fluorescence intensity) are similar in Th1-, Th2-, Th17-polarized and Th0 lymphocytes (Supplementary Figure S7 for a representative example). To note that a subset of cells shows a higher intracellular CD26 intensity, which can be seen in all polarizing conditions, even in non-blasts as soon as they express CD45R0 (data not shown).

### 3.6. sCD26 in the Secretome of In Vitro Polarized CD4 T Lymphocytes

**Figure 7:**
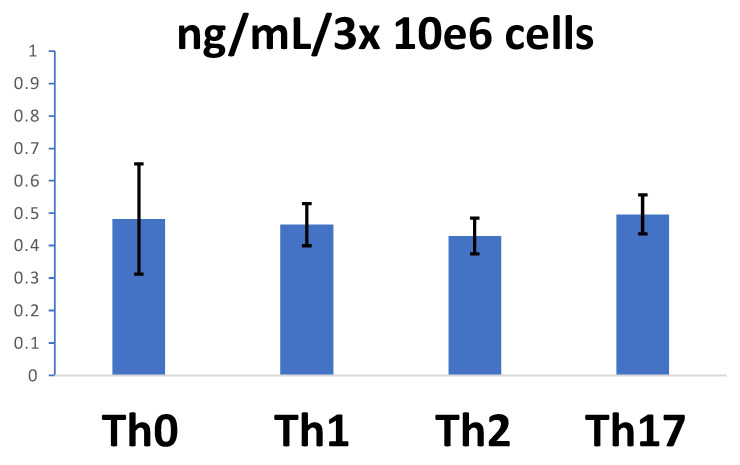
Each bar shows the mean ± SD of ng mL^−1^/3 × 10^6^ cells cultured for 3 days in the following conditions: Th0 (no polarization), Th1, Th2, and Th17 (*n* = 4 or more for each condition).

Important levels of sCD26 in the culture medium, around 40 ng/mL, are found after 3-day culture of 3 × 10^6^ cells/mL in polarization conditions. The mean concentration of sCD26 obtained in the four experiments was similar in the three polarized conditions and Th0 (Figure 7). 

Table 1 shows, however, that the levels, if compared with the Th0 counterpart in each experiment, were usually lower in the secretomes of polarized cells.

This result suggests that the differences in cell-surface CD26 are not explained by changes in the shedding of CD26 from the membrane and, in addition, that the polarizations may alter the levels of circulating sCD26 in the longer term.

## 4. Discussion

In antigen-driven differentiation of naïve CD4 T cells into mature effector T cells, the function of additional activation molecules (Actags, activation antigens) such as CD69, only expressed during the acute period after stimulation are better understood [42] than Actags such as CD26 or CD44, that are also expressed in non-primed naïve T cells and are found soluble in many biological fluids. Until recently, reports on CD26 in the immune system described properties from the population expressing high levels of CD26 and only present in the CD4 CD45R0 subset [3,4,5,8,9]. This isoform of the protein tyrosine phosphatase CD45 is the most used marker of effector/memory cells. Both proteins were supposedly upregulated and associated in the activated T cells [3,4,5,11,12,18]. With an approach like that of Krakauer et al. [4], considering the main distinction between naïve and antigen-experienced CD4 T cells, the first predominantly CD45R0− CCR7+ CD62L+ (L-selectin) and the second predominantly CD45R0+ CD4 T cells, we show that in the CD4 memory/effector subset there are actually more CD26neg than CD26high cells, contrary to the established idea. As most naïve T cells are CD26+, together with the fact that umbilical cord blood lymphocytes and thymocytes are mostly CD26+ [11,12], the CD26neg cells would be originated from CD26neg naïve CD4 cells or, alternatively, the CD26 gene expression would be repressed during some type of differentiation. Our results fit with the latter hypothesis because not only the naïve T CD4 CD45RA but also the CD45R0low cells are basically CD26+.

Bailey et al. [1] also used CD26 to characterize T helper subsets with distinct immunological properties but didn’t use the isotype CD45R0. We further profiled the experienced CD4 CD45R0 T cells subset into central memory cells (T_CM_, CCR7+), which are home to secondary lymphoid organs, and effector memory cells (T_EM_, which have lost CCR7 and are heterogeneous for CD62L) that are home to sites of inflammation [37]. In CD27, a co-stimulatory molecule, expression is also lost in a percentage of T_EM_ with high effector function [37]. We confirmed that CD26high cells are mostly T_EM_, although there is an important CD26neg T_EM_ population (both with variable or negative expression of CCR7, CD62L and CD27). However, more CD26neg cells are related with the T_CM_ population CCR7+ CD27+ CD62L+ (although some T_CM_ are CD26+).

We took advantage of specific adhesion molecules and chemokine receptors expressed by the T cells [1,2,37] for a deeper analysis of T_CM_ and T_EM_ subsets. Circulating non-polarized T_CM_ express CXCR5 and are mainly found in B cell follicles and tonsils. A sizeable proportion (but not all) are CD26neg in accordance with the above results. T_CM_ representing pre-effector cells (pre-Th1 and pre-Th2) express CXCR3 and CCR4, respectively [37]. We show CD26neg cells with expression of these receptors whereas other of these pre-effector cells express CD26, perhaps marking a stage when the non-polarized CD26neg become pre-effector and re-express it. CD4 T_EM_ cells (CXCR5-) that are CD26neg can be observed too, some expressing CCR5+ (specific of Th1 cells) and/or CXCR3 (also in Th2 cells) [37].

On the other hand, only around 50% of CD26high (nearly all CXCR5-) cells express the Th1 markers CCR5 or CXCR3. Together with the presence of CCR4+ CCR5- cells, this all in all confirms the existence of a CD26high population of Th2 phenotype. CCR4 is also expressed on Th17 and Th22 cells, but their frequencies in the whole PBMC are very low to count in this analysis. However, mucosal-associated invariant T (MAIT) cells, representing up to 10% of circulating human T cells, are also CD4 CD26high (there are also CD8 MAIT) and CCR4+ in addition to CD161+ [43,44]. We did not include the CD161 marker in this context, so we could not differentiate between both subsets.

The Th17 or Th22 lineages are almost exclusively CCR6+ [37]. The Th22 cells express the additional chemokine receptors CCR4 and CCR10 [38,39,40,41], and Th17 cells express CD161 in addition to CCR4 [7,10]. We found that Th22 cells are mostly CD26neg and can be excluded from this analysis. However, although Th17 cells were recently reported to be CD26high [1,10], we found that these cells are CCR6+ and CD161+, but CCR4-. A recent report [2] has confirmed this subset CD4 CCR6+CD161+CCR4- CD26high, which elicited the most potent antitumor activity, and probably corresponds with the described non-secreting TGF-beta Th17 cells as different to the Th17 CCR4^+^ subset, which is mostly CD26neg and secrete TGF-beta [36], or as a hybrid Th1/Th17 subset [5].

The well-defined Treg CD25high CCR4high CD26neg subset [16] was also clearly observed in our results, although quantitatively is a minor part of the CD26neg contingent. We observed Tregs, according to CD25 overexpression, that were CD26+ (never CD26high). This finding is interesting because CD26 was once described in clones of Treg cells then called Tr1 and now identified as adaptive iTreg, a distinct population from nTreg, that develop and function in response to pathological situations such as cancer [35,45,46,47,48]. A subset of iTreg expressing ectonucleotidases CD39 and CD73 is able to hydrolyze tATP to 5′ -AMP and adenosine (ADO) and thus mediate suppression of those immune cells which express ADO receptors [45], and CD26 is the ADO deaminase binding protein [18], so it might define a different stage or a different subset in this immunosuppressive environment [48].

Summarizing from the phenotypic observations, naïve T CD4 cells are mostly CD26+ and after T cell activation, in Th1 and Th2 polarization but also in regulatory subsets such as Tregs, Th17 (these ones divided in CCR4+ and -) and Th22, some cells become CD26neg and others CD26high. This landscape is totally different from the role of CD26 as an Actag expressed only in activated T cells in correlation with CD45R0 (probably resembling our data of CD26high cells in Th1 polarizations), suggesting a tight regulation of the surface presence of CD26 within the dynamics of the immune system activity. These results were supported with the in-vitro results, as polarization conditions proved that naïve CD4 T cells directed into the Th1, Th2, or Th17 phenotypes showed strong differences in cell surface CD26 density with respect to the Th0 conditions. CD26 density has been recently associated with the strength of the helper cells to produce cytokines, contributing perhaps in part to explain the differences seen with the ex-vivo results [49], although CD26neg also secrete them [1]. As CD26 is also involved in the role of lipid rafts [50] and in the activation of T cells [6], those studies should be re-evaluated taking into account the presence of T cell subsets with CD26neg, CD26+, and CD26high expression, because they clearly may have clinical consequences not only in oncology, where CD26high CAR-T cells ablated large human tumors to a greater extent than enriched Th17, Th1, or Th2 cells [1,2].

We observed that intracellular staining didn’t match the cell surface changes, a result coherent with the old finding of an intracellular CD26 pool maintained by continuous translation of CD26 mRNA both in CD26+ as well as in CD26neg T lymphocytes [51,52]. Note, however, that recent results suggest that CD26 mRNA can be regulated in certain conditions [53], and the promoter of the human *DPP4* gene contains consensus sequences for transcription factors such as NF-kappa B [54] or STAT1 [55], with key roles in the immune system. In those early experiments of antigenic modulation with anti-CD26 mAbs, the surface expression of new CD26 was very fast, suggesting a regulation of the mechanisms of translocation of intracellular membrane to the cell surface [51,52]. Whether the recently discussed presence of serum anti-CD26 auto-Abs [56] would have a role on surface CD26 expression is unknown.

An alternative to the regulation of gene expression could be the regulation of the shedding/secretion of cell surface CD26 into the culture medium [17,18,19,20,31]. The activated cells with very high CD26 intracellular expression observed in this work might be related to the secretory lysosomes of some T cells [32,33], and this fact will be studied further.

The concentration of sCD26 found in the supernatants of the cell cultures was qualitatively important, showing that activated CD4 T cells are an obvious source of circulating sCD26 (it can be originated from more tissues) (reviewed in [18,20]). A recent report suggests that immune cells can be responsible of around 75% of circulating sCD26 [17].

However, the three polarizations showed similar results, a small decrease in the presence of the soluble form, perhaps by impairing the secretory subset. The decrease was not quantitatively relevant in the short term but suggests that the maintenance of these T cell responses in the long term might be responsible, at least in part [32,33], of the different levels of circulating sCD26 found in different diseases [17,18,20,57,58,59]. Some functions of CD26 are different in mice and human, so additional models to further study this point should be carefully considered.

The functions already proposed for sCD26, inducing human neutrophil chemorepulsion [22,23,24] or proliferation of human smooth muscle cells [28] have shown clinical consequences in inflammatory diseases including infection, autoimmune [22,23,24], and probably cancer [25,26,27], so clarifying the relationship between the CD26 expression on lymphocytes and sCD26/DPP4 may help to advance in the knowledge of their physiological roles and therapeutic approaches [18,19,20,34,35,57,58,59,60,61,62].

## Figures and Tables

**Table 1:** Differences in culture medium sCD26 levels after T lymphocyte polarization with respect to nonpolarizing activation conditions in four donors *.

	Polarization Condition		
	Th1	Th2	Th17
	−9	−11	−18
**Soluble CD26 (sCD26)**	−13	−15	−17
	−4	−14	10
	−20	−22	-

* Data shown are the percentages’ differences (%) between sCD26 levels from each T helper polarization condition in comparison to the non-polarizing Th0 condition used as control in four experiments. After 72 h of stimulation as described in methods, cells were collected by centrifugation and culture supernatants stored at −20 °C for use in subsequent sCD26 determination with the human DPPIV/CD26 DuoSet ELISA development System kit (RnD Systems) according to the manufacturer’s instructions. In this way, the ANOVA for the 4 conditions was close to significance (*p* = 0.055) and the post-hoc analysis showed that the statistically different group was the Th2 group.

## Data Availability

The datasets analyzed during the current study are available from the corresponding author on reasonable request.

## Supplementary Materials

The following are available online at https://www.mdpi.com/article/10.3390/biom11101446/s1, Figure S1: Isotype controls for the flow cytometry staining of Ficoll-purified PBMC. Figure S2: Major CD4+ T cell subsets defined by surface CD45R0 and CD26 expression. Figure S3: Surface CCR7, CD62L and CD27 positivity frequencies in the major CD4^+^ T cell subsets defined by surface CD45R0 and CD26 expression. Figure S4: Surface CD25 expression to define T_reg_ subsets defined by CD26 expression. Figure S5: Surface CXCR5, CCR4, CXCR3 and CCR5 positivity frequencies in the major CD4^+^ T cell subsets defined by surface CD45R0 and CD26 expression. Figure S6: Gating strategy for lymphocytes after the in-vitro activation in polarizing or not conditions. Figure S7: Intracellular CD26 expression in CD45R0+ CD4 T cells (blasts) activated in vitro under different polarization conditions.
