# Supplementary material for: Distinctive CD26 Expression on CD4 T-Cell Subsets
Source: Biomolecules. 2021 Oct 2;11(10):1446. doi: 10.3390/biom11101446 (PMC8533622; doi:10.3390/biom11101446)

Supplementary Figure S1. Isotype controls for the flow cytometry staining of Ficoll-purified PBMC. Lymphocytes were gated on FSC and SSC, and the dot plots (vs SSC) show the staining with isotype IgG1 or IgG2 controls labelled with the indicated fluorochromes.

Supplementary Figure S2. Surface CCR7, CD62L, CD27, CXCR5, CCR4, CXCR3 and CCR5 expression on Ficoll-purified PBMC. Dot plots show the staining of the anti-CD45R0 mAb vs the staining of the mAb against each marker (with the fluorochromes shown) on CD4 T cells.

Supplementary Figure S3. Surface CCR7, CD62L and CD27 positivity frequencies in the major CD4$^+$ T cell subsets defined by surface CD45R0 and CD26 expression. To analyze the presence of CD26 in defined T_{EM} and T_{CM}**,** lymphocytes are gated using the same strategy shown in Figure 1, (A) CD26neg cells are shown in red, CD26+ (intermediate) cells are shown in black and CD26high cells are shown in blue. Representative histograms showing the expression of B) CCR7, C) CD62L and D) CD27 in CD4+ CD45R0+ T lymphocytes with different levels of CD26 are shown.

Supplementary Figure S4. Surface CD25 expression to define T_{reg} subsets defined by CD26 expression. Left panel) Lymphocytes are gated using the same strategy shown in Figure 1. Right panel) Representative histogram showing the expression of different levels of CD26. CD26neg cells are shown in red, CD26+ (intermediate) cells are shown in black and CD26high cells are shown in blue.

Supplementary Figure S5. Surface CXCR5, CCR4, CXCR3 and CCR5 positivity frequencies in the major CD4$^+$ T cell subsets defined by surface CD45R0 and CD26 expression. To analyze the presence of CD26 in different T_{EM} and T_{CM} populations, lymphocytes are gated using the same strategy shown in Figure 1 and shown in Sup Fig 2. CD26neg cells are shown in red, CD26+ (intermediate) cells are shown in black and

CD26high cells are shown in blue lines. Representative histograms showing the expression of A) CXCR5, B) CCR4, C) CXCR3 and D) CCR5 in CD4+ CD45R0+ T lymphocytes with different levels of CD26 are shown.

Supplementary Figure S6. Gating strategy for lymphocytes after the *in-vitro* activation in polarizing or not conditions. On the left panel, the total lymphocyte population was physically gated, and the isotype **c**ontrols without (left column) and with (right column) gating on FSC/SSC are shown. The dot plot on the right shows the CD3/CD4 staining of the gated lymphocytes, corresponding to the CD26/CD45R0 staining shown in the Figure 6.

Supplementary Figure S7. Intracellular CD26 expression in CD45R0+ CD4 activated T cells (blasts) activated *in vitro* under different polarization conditions. Representative dot plots of gating and intracellular CD26 expression in Th0 and Th2 as examples. Cells shown are those gated on CD45R0 positive cells (top left panel, gate).

Sup Figure S1 Purified blood lymphocytes gated on FSC and SSC. Dot plots show isotype controls with fluorochromes:

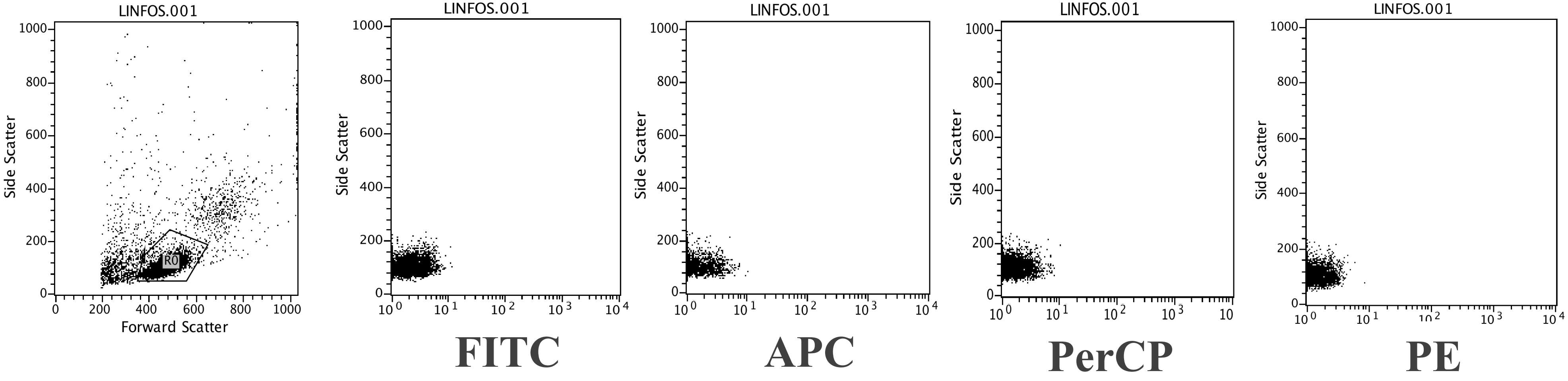

Sup Figure S2 Gated on FSC and CD4, dot plots show CD45R0 staining vs markers of:

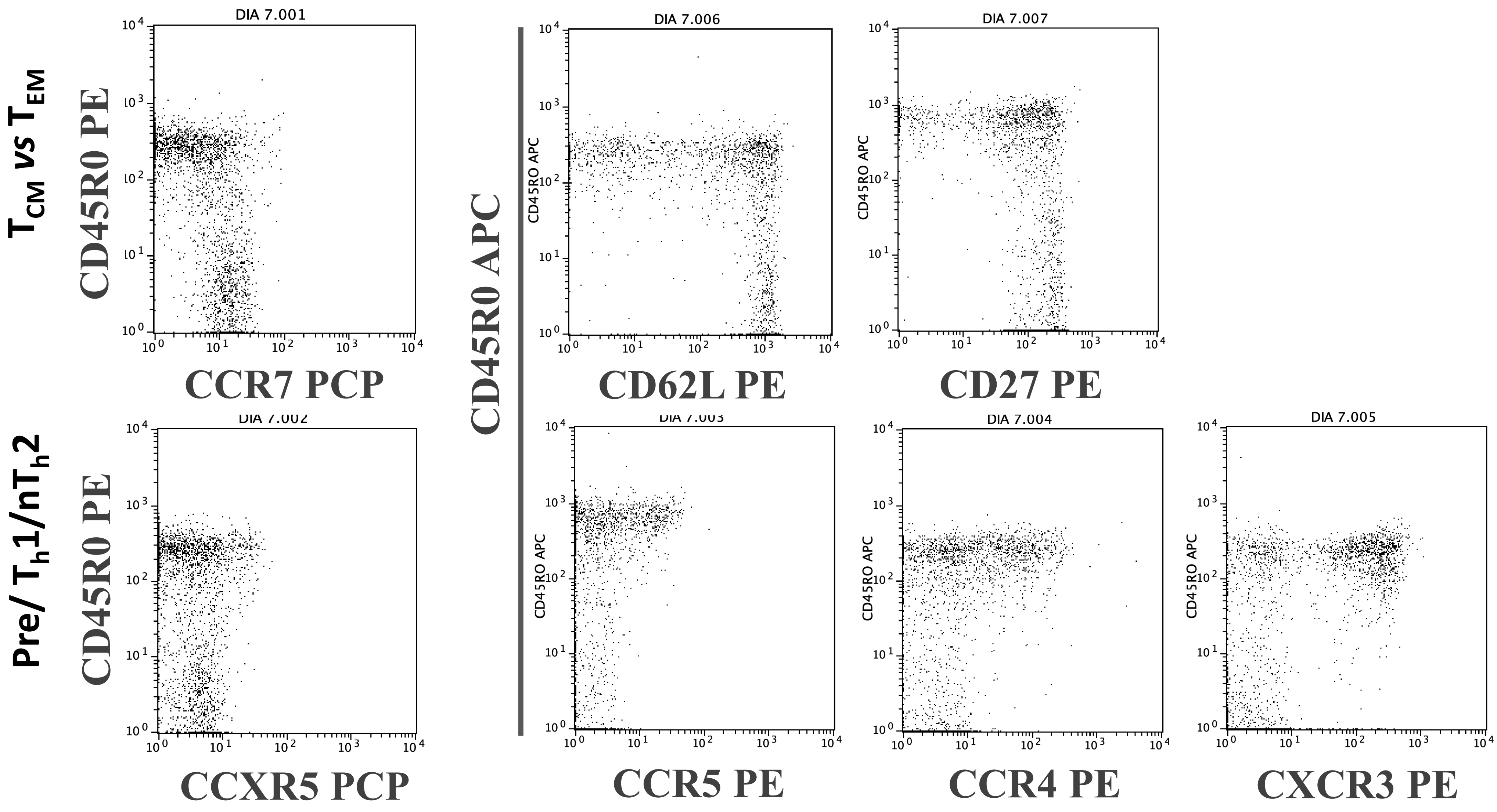

Sup Figure S3

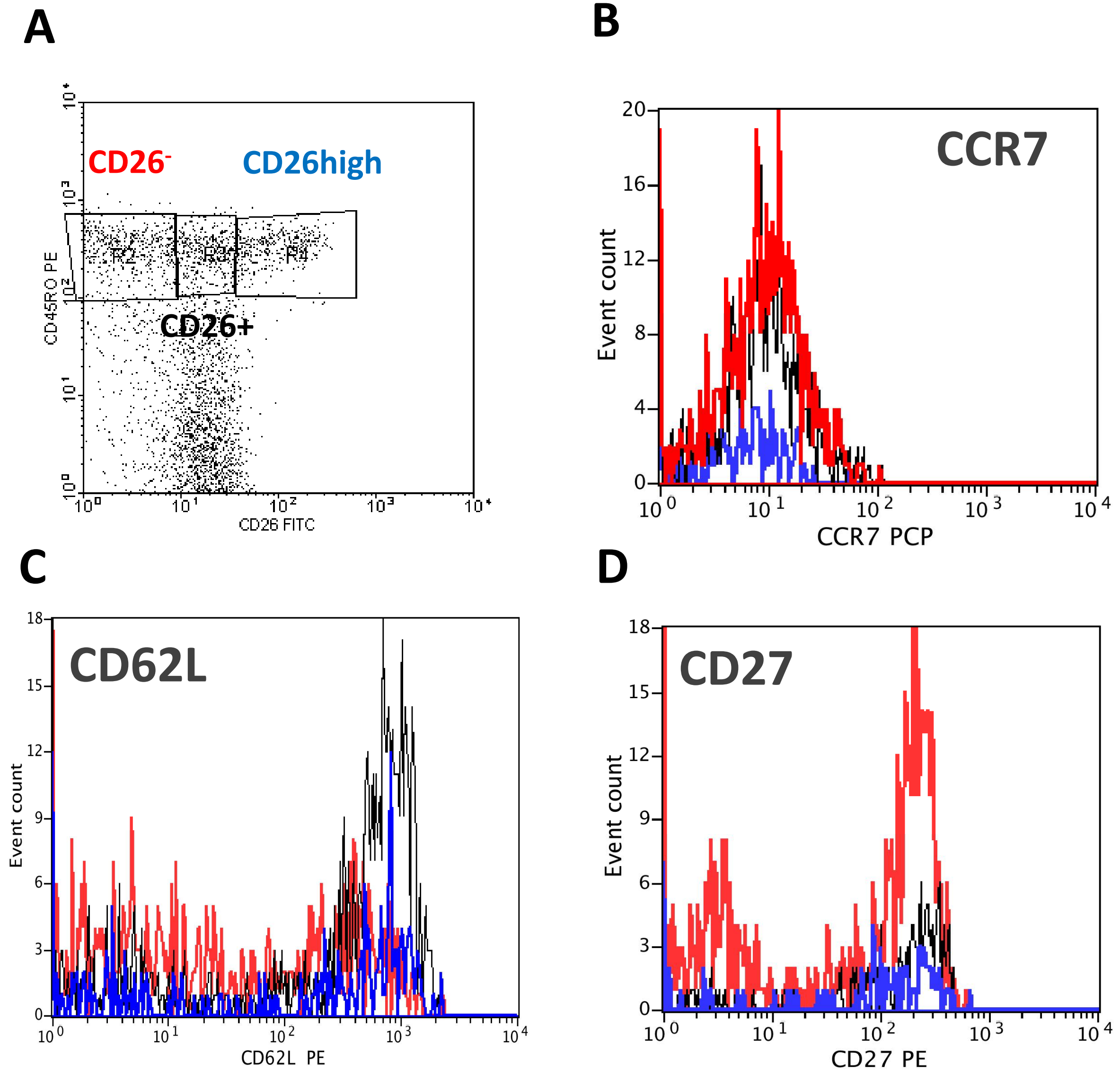

Sup Figure S4

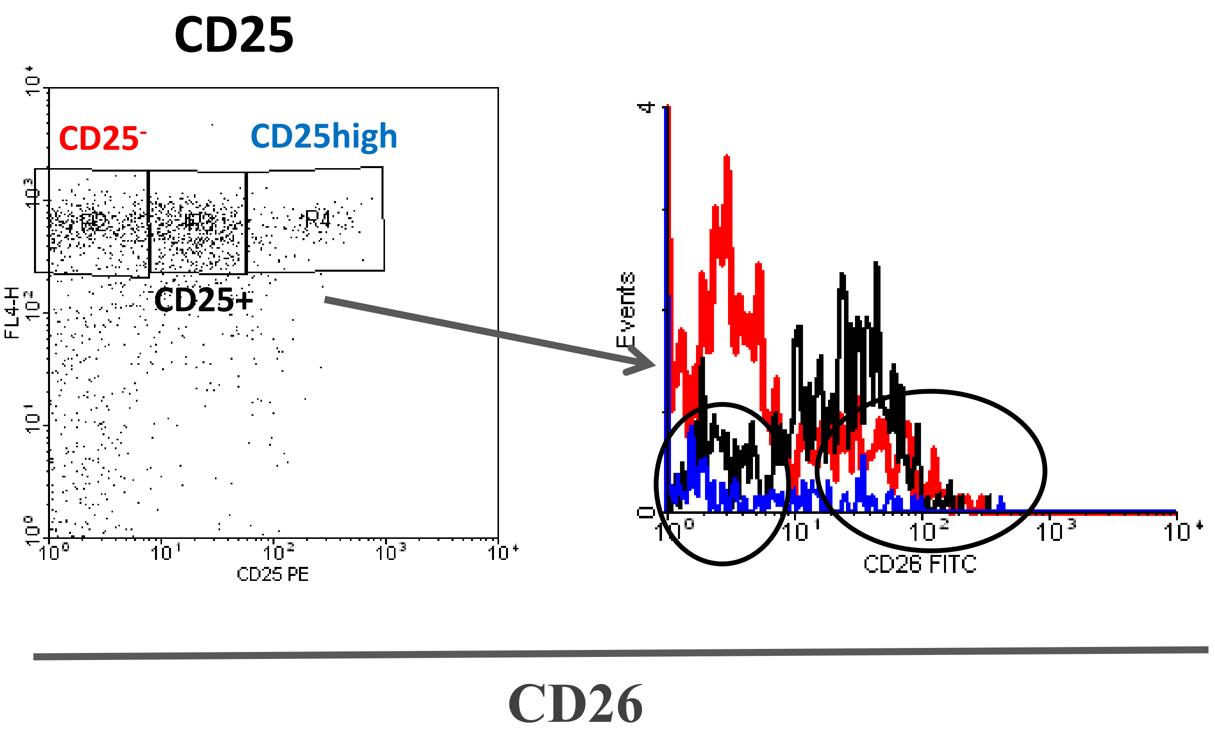

Sup Figure S5

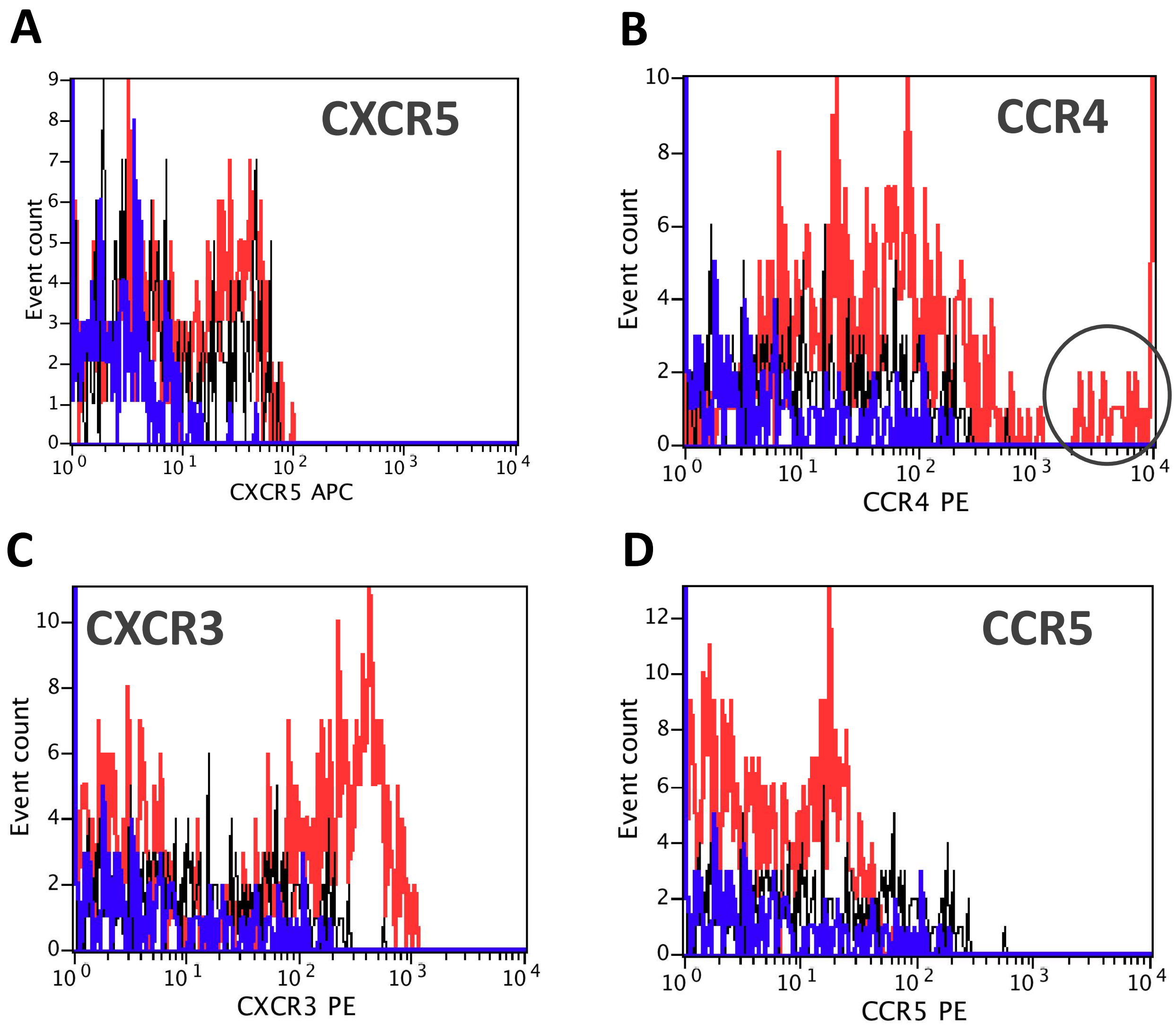

Sup Figure S6

Gatings of lymphocytes after *in-vitro* activation:

Controls without (left) and with (right) gating on FSC/SSC

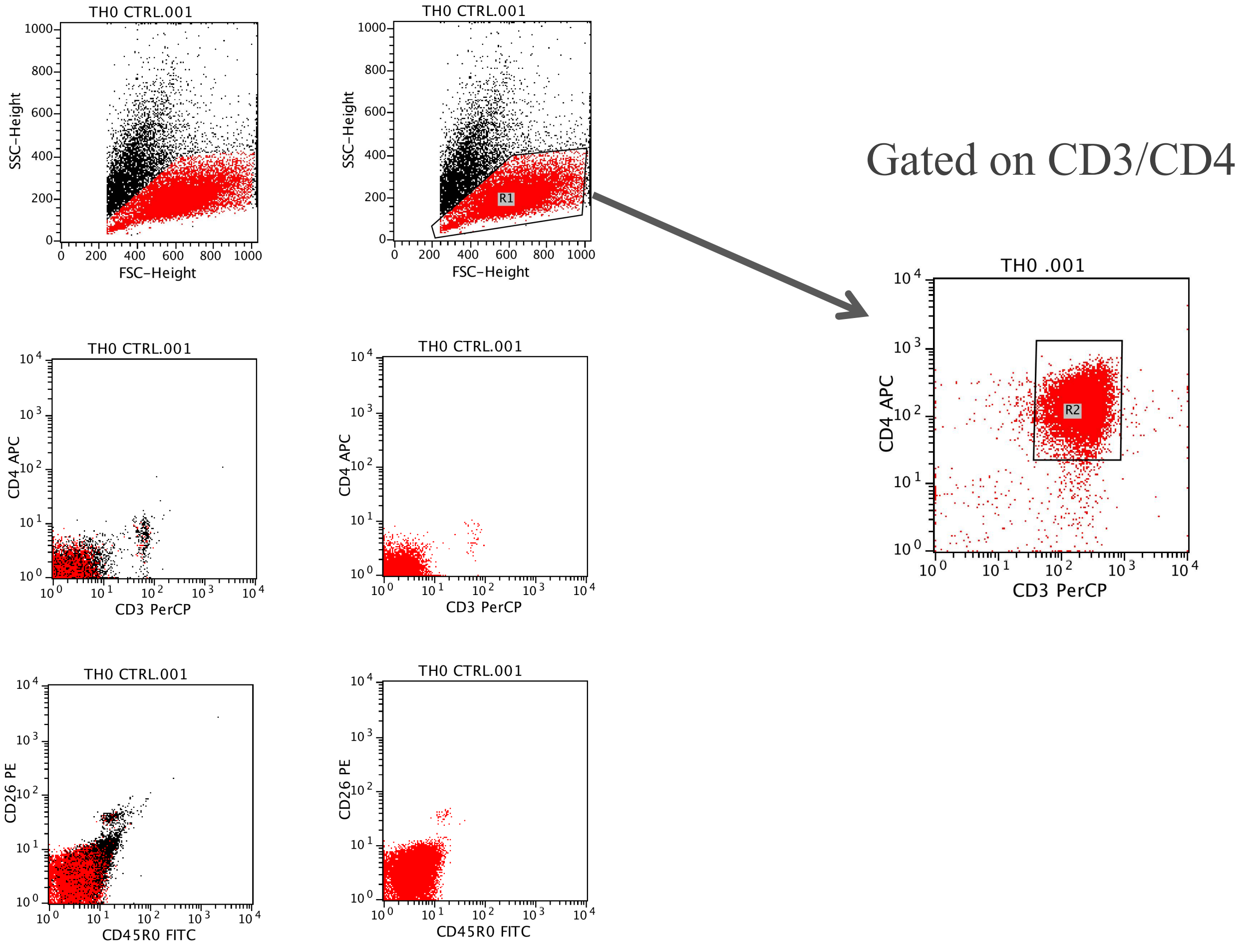

Sup Figure S7

Gated on FSC and CD45R0 after activation. Intracellular staining of:

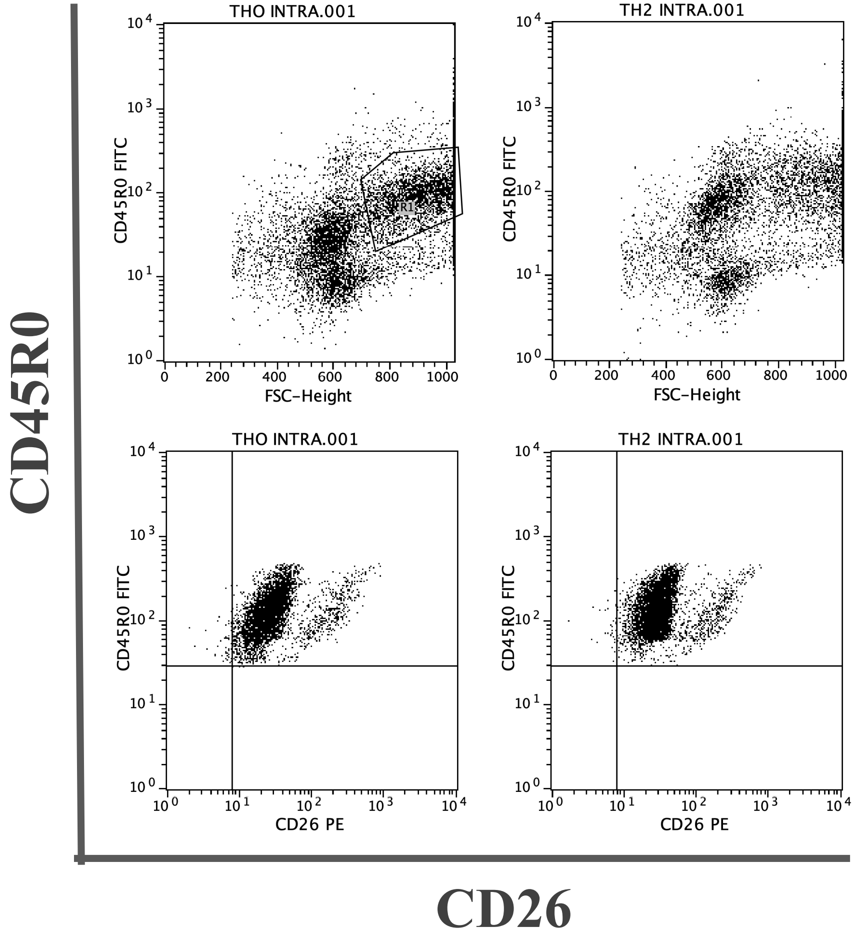

Supplement: Supplementary file 1 [file biomolecules-11-01446-s001.zip › biomolecules-1314214-supplementary.pdf]
